# Triage Procedures for Critical Care Resource Allocation During Scarcity

**DOI:** 10.1001/jamanetworkopen.2023.29688

**Published:** 2023-08-29

**Authors:** Jackson S. Ennis, Kirsten A. Riggan, Nicholas V. Nguyen, Daniel B. Kramer, Alexander K. Smith, Daniel P. Sulmasy, Jon C. Tilburt, Susan M. Wolf, Erin S. DeMartino

**Affiliations:** 1 Biomedical Ethics Research Program, Mayo Clinic, Rochester, Minnesota; 2 Richard A. and Susan F. Smith Center for Outcomes Research in Cardiology, Beth Israel Deaconess Medical Center, Harvard Medical School, Boston, Massachusetts; 3 Harvard Medical School Center for Bioethics, Boston, Massachusetts; 4 Department of Medicine, Division of Geriatrics, University of California, San Francisco; 5 San Francisco Veterans Affairs Medical Center, San Francisco, California; 6 Departments of Medicine and Philosophy, Georgetown University, Washington, DC; 7 Kennedy Institute of Ethics, Georgetown University, Washington, DC; 8 Division of General Internal Medicine, Mayo Clinic, Scottsdale, Arizona; 9 University of Minnesota Medical School, Minneapolis; 10 University of Minnesota Law School, Minneapolis; 11 Division of Pulmonary and Critical Care Medicine, Mayo Clinic, Rochester, Minnesota

## Abstract

**Importance:**

During the COVID-19 pandemic, many US states issued or revised pandemic preparedness plans guiding allocation of critical care resources during crises. State plans vary in the factors used to triage patients and have faced criticism from advocacy groups due to the potential for discrimination.

**Objective:**

To analyze the role of comorbidities and long-term prognosis in state triage procedures.

**Design, Setting, and Participants:**

This cross-sectional study used data gathered from parallel internet searches for state-endorsed pandemic preparedness plans for the 50 US states, District of Columbia, and Puerto Rico (hereafter referred to as states), which were conducted between November 25, 2021, and June 16, 2023. Plans available on June 16, 2023, that provided step-by-step instructions for triaging critically ill patients were categorized for use of comorbidities and prognostication.

**Main Outcomes and Measures:**

Prevalence and contents of lists of comorbidities and their stated function in triage and instructions to predict duration of postdischarge survival.

**Results:**

Overall, 32 state-promulgated pandemic preparedness plans included triage procedures specific enough to guide triage in clinical practice. Twenty of these (63%) included lists of comorbidities that excluded (11 of 20 [55%]) or deprioritized (8 of 20 [40%]) patients during triage; one state’s list was formulated to resolve ties between patients with equal triage scores. Most states with triage procedures (21 of 32 [66%]) considered predicted survival beyond hospital discharge. These states proposed different prognostic time horizons; 15 of 21 (71%) were numeric (ranging from 6 months to 5 years after hospital discharge), with the remaining 6 (29%) using descriptive terms, such as long-term.

**Conclusions and Relevance:**

In this cross-sectional study of state-promulgated critical care triage policies, most plans restricted access to scarce critical care resources for patients with listed comorbidities and/or for patients with less-than-average expected postdischarge survival. This analysis raises concerns about access to care during a public health crisis for populations with high burdens of chronic illness, such as individuals with disabilities and minoritized racial and ethnic groups.

## Key Points

**Question:**

How are comorbidities and prognosis relevant to state-level critical care triage procedures at times when resources are scarce?

**Findings:**

This cross-sectional analysis of 32 state-promulgated written triage procedures found that 20 states listed comorbidities that would restrict or preclude access to scarce resources, although conditions listed and proposed severity assessments differed by state. Twenty-one states also conditioned access on an estimation of length of life after hospital discharge.

**Meaning:**

In this study, comorbidities and prognosis beyond hospital discharge frequently factored into triage procedures, restricting individuals from accessing critical care under conditions of scarcity in a pandemic.

## Teaser

This cross-sectional study analyzes the role of comorbidities and long-term prognosis in state triage procedures.

## Introduction

In early 2020, as concerns grew over projected shortages of critical care resources during the COVID-19 pandemic, many US states developed or revised plans for the allocation of scarce critical care resources.^1,2^ Some state-level pandemic preparedness plans (PPPs) had initially been developed during the 2009 H1N1 pandemic, based on recommendations from the Institute of Medicine (now National Academy of Medicine) for states to provide instruction for allocating scarce critical care resources.^3^ These PPPs ranged from broad statements of ethical principles to detailed clinical instructions for triage, while some states issued no guidance.

Elements of many states’ PPPs provoked concerns for potential discrimination early in the COVID-19 pandemic, particularly among disability advocates, older adults, and civil rights groups.^4,5,6^ Most states’ triage procedures relied on scoring systems to classify the degree of organ dysfunction, most commonly the Sequential Organ Failure Assessment (SOFA).^7^ Critics argued that SOFA was not validated for respiratory illness, including COVID-19; inaccurately predicted mortality; and perpetuated algorithmic bias against individuals with chronic conditions, notably Black patients with chronic kidney disease and persons with motor or verbal impairments, because the Glasgow Coma Scale is used in the SOFA score.^7,8,9,10,11,12^ In response, many states modified SOFA scoring systems to mitigate these shortcomings.^7^ Additionally, the Department of Health and Human Services Office for Civil Rights (OCR) worked to resolve complaints of discrimination against at least 5 states’ PPPs through 2020 and 2021. To comply with federal antidiscrimination laws, pursuant to Section 504 of the Rehabilitation Act and Section 1557 of the Patient Protection and Affordable Care Act, OCR instructed states to avoid categorical exclusion criteria and factoring long-term survival or required resource intensity into triage.^13,14,15^

Despite efforts from civil rights groups, the OCR, and patient advocates, as well as revision by many states, ethical and legal concerns persist concerning discriminatory provisions in PPPs. While earlier studies have examined how age, disability, and SOFA factor into states’ triage procedures,^16,17,18^ no study has systematically examined how prognostic assessments of posthospital survival factor into triage. This is a key area of clinical, ethical, and legal concern. This analysis sought to answer the question of how comorbidities and postdischarge survival factor into US states’ triage procedures, hypothesizing that more than 3 years into the pandemic, state-promulgated triage plans that restrict access to critical care resources on the basis of these patient factors would persist.

## Methods

### Identifying Pandemic Preparedness Plans

Two members of the review team (among J.S.E., K.A.R., N.V.N., and E.S.D.) performed independent internet searches for PPPs for 52 US jurisdictions (US states, Washington DC, and Puerto Rico, hereafter referred to as states) that were publicly available between November 25, 2021, and June 16, 2023. The first 20 results of a Google search were reviewed after inserting each state’s name and a standard list of terms, including *crisis standards of care*, *resource allocation*, *ventilator allocation*, *rationing protocols*, *triage guidelines*, *pandemic influenza plan*, and *emergency operations plan*. If no relevant plan related to critical care triage was identified, the state’s department of health website was searched. Search findings were compared with existing publications on state-level PPPs.^16,17,18^ For states where no PPP was available or state endorsement was unclear, the team contacted the state health department. Box 1 contains further descriptions of the terms used in this analysis. As the data collected were publicly available and did not involve human participants or animal subjects, no institutional review board review was required per the Common Rule. This study follows the relevant portions of Strengthening the Reporting of Observational Studies in Epidemiology (STROBE) guidelines for cross-sectional studies.^19^

Box 1. **Terms Relevant to This Analysis, Including Descriptions and Examples**

**State Pandemic Preparedness Plans**

State-promulgated document(s) for navigating public health crises, including strategies for allocating scarce resources. In many, but not all, states these documents are labeled *crisis standards of care*. Some states have issued a series of guidance documents, ranging from ethical guidance to clinical protocols for allocating specific resources.

**Triage**

The process by which patients presenting for care are prioritized to receive scarce hospital or critical care resources. Triage sometimes follows a tiered workflow, whereby primary triage criteria are applied to all patients and secondary triage criteria are applied to a subset.

**Written Triage Procedures**

A subset of pandemic preparedness plans that offer detailed instructions to direct allocation of scarce hospital or critical care resources. Priority scores are typically assigned based on a severity of organ dysfunction assessment (such as SOFA), with further points assigned for diseases or prognosis.

**Triage Officers**

Individual(s) responsible for conducting triage. Most states with triage protocols advise that triage officers be separate from the clinical team caring for patients. Depending on setting, triage could be performed by a single individual or a triage team. Some states provide instructions for the composition of the triage team, while others are ambiguous.

**Comorbid conditions and comorbidities**

Chronic conditions or conditions coincident to the presenting illness or injury (eg, advanced cirrhosis in a patient presenting with subarachnoid hemorrhage).

**Prognostication Beyond Hospital Discharge**

Instructions for triage officers to estimate duration of survival beyond hospital discharge (eg, 6 mo or 5 y after discharge; also short-term and long-term), based on the presence and/or severity of comorbid conditions or global assessment of the patient’s state of health.

**Exclusion Criteria**

Conditions considered during primary triage that exclude the patient from accessing resources.

**Deprioritization Criteria**

Conditions considered during primary triage that result in the patient receiving lower priority for accessing resources.

**Tiebreaker Criteria**

Clinical or contextual factors used in a secondary stage of triage to determine which patient should receive a resource when multiple patients have equivalent triage scores (eg, pregnancy or essential worker status as tiebreakers).

Abbreviation: SOFA, Sequential Organ Failure Assessment.

### Data Analysis

#### Primary Analysis

Three review team members (among J.S.E., K.A.R., N.V.N., and E.S.D.) independently reviewed documents identified by the previously described multiphase search and publicly available on the search end date, June 16, 2023. The team met weekly to generate consensus on inclusion criteria and inductive coding categories. Only documents that were publicly available during the search interval and hosted on state government websites or endorsed by government entities (as evidenced by the state’s department of health seal or a cover letter displaying state officials’ names) were included. Documents marked as draft or issued by nongovernmental entities (eg, hospital associations) without state endorsement were excluded. Documents offering only ethical guidance and lacking step-by-step triage instructions to direct allocation of scarce critical care resources in clinical practice were also excluded. If multiple PPPs were identified for a single state, only the most recent document was analyzed. Allocation frameworks for vaccines and COVID-19 therapeutics were not included.

#### Secondary Analysis

Next, triage procedures with step-by-step instructions for prioritizing patients were identified, as these would likely inform clinical practice across health systems within a given state. States’ use of comorbid medical conditions in these triage procedures were analyzed, tabulating any disease-specific measures of severity proposed by states. Each state’s written triage procedure was categorized based on the inclusion of a list or lists of comorbidities, and if present, the list’s function in triage (exclusion, deprioritization, or tiebreaker). Any provisions restricting access based on predicted length of survival beyond discharge were analyzed, including the prognostic range (number of months to years or descriptions such as long-term). Finally, the resource allocated by the written triage procedure was categorized.

The review team reached consensus on the categorization of the examined features of written triage procedures. The final analytic framework was applied to all triage procedures that were publicly accessible on June 16, 2023.

## Results

Of 44 state pandemic preparedness plans identified, 32 met inclusion criteria. Twenty-seven triage procedures (84%) restricted access to scarce critical care resources for patients with listed medical conditions or for patients with limited predicted postdischarge survival.

### Identification of Written Triage Procedures

**Figure 1.**
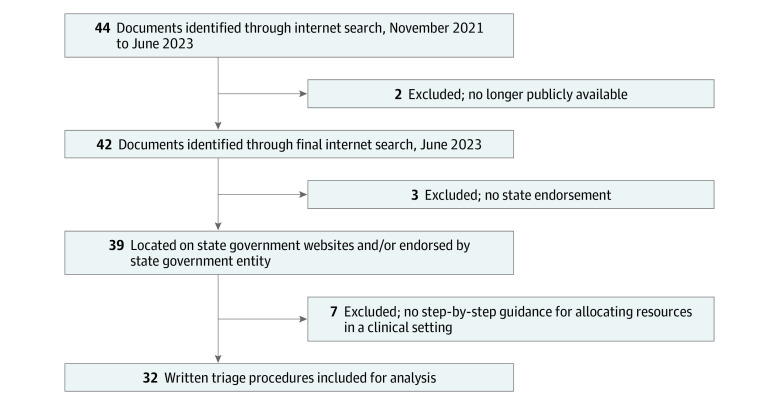
Identifying States’ Written Triage Procedures Internet searches for states’ pandemic preparedness plans were conducted; eligible documents were accessed through state government websites or had documentation of state government endorsement. Policies were excluded from analysis if they offered only general ethical guidance and did not contain written triage procedures. Overall, 32 states had publicly available, state-endorsed written triage procedures.

Forty-four state PPPs were identified across the 52 states (Figure 1). Documents were excluded if they were no longer publicly available in June 2023 (2), lacked state endorsement (3), or did not contain written triage procedures for clinical practice (7). Thirty-two documents met inclusion criteria and underwent further analysis. eTable 1 in Supplement 1 includes web links to documents and state-level PPP details.

During the search period, 12 states updated or removed their plans. Two states with written triage procedures including lists of comorbidities and prognostication beyond hospital discharge (Hawaii and North Carolina) removed their PPPs from public access before our end date of June 16, 2023, and were not included in this analysis. Ten states (Colorado, Idaho, Kansas, Michigan, Mississippi, Montana, New Hampshire, Oregon, South Dakota, and South Carolina) revised triage procedures; the most recent documents from these states were included.

### Lists of Comorbid Conditions

**Figure 2.**
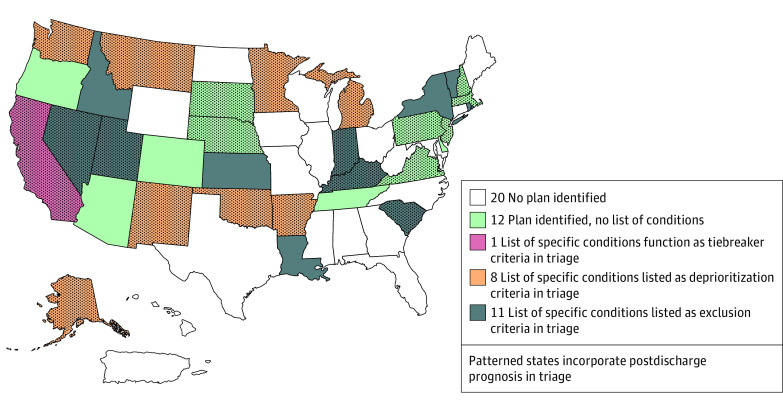
States Factoring Comorbidities and Postdischarge Prognosis Into Triage In state triage procedures, lists of conditions played different functions. Eleven states’ triage protocols excluded patients with particular comorbidities (exclusion criteria). Eight states deprioritized patients with particular comorbidities during triage (deprioritization criteria); 1 state listed particular conditions to be considered as a tiebreaker, if more than 1 patient received the same triage score. Kansas, an exclusion state, also included a list of deprioritizing conditions in its Chronic Advanced Organ Dysfunction Score, a component of triage score assignment. Twelve states had written triage procedures that did not list comorbidities that would restrict patients’ access to critical care. Twenty-one states’ written triage procedures incorporated postdischarge prognosis into triage. Twenty jurisdictions, among them Puerto Rico and the District of Columbia, either did not issue pandemic preparedness plans or their plans lacked written triage procedures.

Of the 32 states with written triage procedures, 20 (63%) included lists of comorbid conditions (Figure 2). Under these policies, patients with listed conditions would be categorically excluded from access to the scarce resources being triaged in 11 of 20 states (55%). In 5 of these 11 states (45%; ie, Utah, Idaho, Kansas, New York, and Rhode Island), the listed conditions would result in near-immediate mortality (eg, cardiac arrest unresponsive to resuscitation protocols, severe trauma unlikely to result in survival with unlimited resources). Eight of 20 states (40%) included lists of conditions that, if present, would deprioritize an individual in triage relative to other patients. In addition to their exclusion criteria, Kansas created a Chronic Advanced Organ Dysfunction Score, deprioritizing patients by assigning additional triage points on the basis of listed chronic illnesses. No other state included more than 1 list of conditions with different functions within triage. Finally, California’s list of conditions functioned as a tiebreaker, differentiating between patients with equal triage scores. Sixteen of the 20 state lists of comorbidities (80%) explicitly granted triage officers permission to exercise clinical judgement and consider additional conditions of similar severity that were not listed (eTable 1 in Supplement 1). Twelve of the 32 states with written triage procedures (38%) did not include a list of conditions.

### Proposed Severity Assessments of Comorbidities

Box 2 organizes comorbidities by organ system and includes examples of states’ proposed severity assessments for use in triage (a complete tabulation can be found in eTable 2 in Supplement 1). The most frequently listed conditions were malignant neoplasm (13 of 20 [65%]), heart failure (13 of 20 [65%]), cirrhosis (13 of 20 [65%]), and chronic lung disease (12 of 20 [60%]). Proposed assessments of severity differed from state to state, both in their specificity and in the severity of disease resulting in exclusion or deprioritization during triage. For example, some states used descriptive phrases for heart failure to indicate severity (eg, “poor prior prognosis”; “evidence of frailty”) while others referenced clinical parameters (eg, ejection fraction 21%-40%; American College of Cardiology/American Heart Association Stage D). Even for a given condition, states proposed a variety of thresholds: in Oklahoma, presence of New York Heart Association (NYHA) Class II heart failure deprioritized patients during triage, whereas in New Mexico deprioritization would occur only for patients with NYHA IV heart failure. During the search period, Montana updated its PPP to include a new list of deprioritizing conditions, making it the only state to include neonatal parameters such as “birth weight <500 grams” and “newborn with gestational age <24 weeks.”

Box 2. **Comorbid Conditions Listed by 20 States as Exclusion or Deprioritization Triage Criteria^a^**

**Neurologic**

- Dementia or Alzheimer disease: FAST score ≥7; “moderate”; “limited speech ability”; “no independent ambulatory ability”; “loss of ability to smile”; hospice-eligible
- Neurodegenerative or neuromuscular disease (eg, ALS, MS, SMA, PD): “advanced, progressive”; “requiring assistance with ADLs or requiring chronic ventilator support”
- Chronic disorder of consciousness: modified Rankin Score ≥5; “persistent vegetative state”
- Neurologic injury or event: “no motor response to painful stimuli”; “high expected mortality”; coma >72 hr; GCS <6; “irreversible”; minimal chance of recovery by neurospecialist’s assessment; “CT evidence of herniation”; massive middle cerebral artery or brainstem stroke; spontaneous massive intracerebral hemorrhage with ICH score >3

**Cardiovascular**

- Cardiac arrest: “unresponsive to standard interventions”; “unwitnessed arrest”; “recurrent arrest”; “trauma-related arrest”
- Hypotension: “unresponsive to fluid resuscitation and vasopressors”
- Pulmonary hypertension: WHO Class 3; WHO Class 4
- Coronary artery disease: “severe multivessel”; “symptomatic, not amenable to treatment”; “multiple stents placed or CABG”
- Heart failure: EF 21%-40%; EF <25%; “persistent ischemia unresponsive to therapy”; “non-reversible ischemia with pulmonary edema”; ACC/AHA stage D; NYHA Class II; NYHA Class III; NYHA Class IV; “frailty”; “poor prior prognosis”

**Respiratory**

- Chronic lung disease: home oxygen dependent; FEV_1_ <20% predicted; FEV_1_ <25% predicted; “frailty”; “poor prior prognosis”; “on NIPPV”
- Obstructive lung disease or COPD: home oxygen dependent; “moderately severe”; “with severe secondary pulmonary hypertension”
- Restrictive lung disease or pulmonary fibrosis: home oxygen dependent; FVC <35% predicted; “moderately severe”; TLC <60% predicted; PaO_2_ <55 mm Hg; severe secondary PH
- Cystic fibrosis: home oxygen dependent; postbronchodilator FEV_1_ <30% predicted

**Gastrointestinal and Hepatic**

- Acute hepatic failure: hyperammonemia
- Cirrhosis: MELD ≥20; presence of “ascites, history of variceal bleeding, coagulopathy or encephalopathy”; “history of decompensation”; Child-Pugh score >9; Child-Pugh Class C; ineligible for transplant; “poor prior prognosis”; MELD >15

**Kidney**

- End stage kidney disease: dialysis dependent; “end-stage renal disease in patients <75”

**Neoplastic**

- Malignant neoplasm: “metastatic”; <10 y survival; “incurable”; ECOG performance status ≥3; palliative treatment only; “unresponsive to interventions”
- Solid organ malignant neoplasm: “poor prognosis for recovery”; “expected survival <6 months despite treatment”; “poor expected response to therapy”
- Hematologic malignant neoplasm: “poor prognosis for recovery”; “resistant or progressive despite conventional initial therapy”

**Injury**

- Severe burn or trauma: “predicted survival ≤10%”; BSA >40%; severe inhalation injury; <50% survival on triage for burn victim assessment; burn center consultation; low likelihood of survival on American Burn Association Guide

**General**

- Hospice eligible
- Immunocompromised: “high short-term mortality”
- Neonatal: “birthweight <500 grams”; “newborn with gestational age <24 weeks”; “bilateral grade 4 intraventricular hemorrhage”; “total bowel loss due to necrotizing enterocolitis”

Abbreviations: ACC, American College of Cardiology; ADLs, activities of daily living; AHA, American Heart Association; ALS, amyotrophic lateral sclerosis; BSA, body surface area; CABG, coronary artery bypass graft; COPD, chronic obstructive pulmonary disease; CT, computed tomography; ECOG, Eastern Cooperative Oncology Group; EF, ejection fraction; FAST, Functional Assessment Staging Tool; FEV_1_, forced expiratory volume in the first second; FVC, forced vital capacity; GCS, Glasgow Coma Scale; ICH, intracerebral hemorrhage; MELD, Model for End-Stage Liver Disease; MS, multiple sclerosis; NIPPV, non-invasive positive pressure ventilation; NYHA, New York Heart Association; PD, Parkinson disease; PH, pulmonary hypertension; SMA, spinal muscular atrophy; TLC, total lung capacity; WHO, World Health Organization.

^a^
Conditions listed in state plans spanned most organ systems, although the most prevalent diseases mentioned were malignant neoplasm, heart failure, cirrhosis, and lung disease. States sometimes proposed assessments of disease severity, which ranged from rigorously studied (eg, ECOG performance status, MELD) to purely subjective (eg, “poor prognosis for recovery,” “loss of ability to smile”). Examples of these proposed severity assessments are provided. A spectrum of disease severity (eg, NYHA Class II, Class III and Class IV heart failure) reflects the fact that different states have different thresholds for what severity of illness results in deprioritization or exclusion during triage.

### Resources Allocated in Triage

Written triage procedures in different states focused on allocating different resources (eFigure in Supplement 1). Of the 32 states with written triage procedures, 11 (34%) indicated their purpose was to allocate a specific resource. These procedures referred most commonly to mechanical ventilation, but sometimes referred simply to a scarce resource. Seventeen of 32 states’ triage procedures (53%) allocated critical care more broadly, often referencing an intensive care unit bed as the resource being allocated. Four of 32 states’ written triage procedures (13%; ie, Kentucky, Louisiana, South Dakota, and Tennessee) allocated hospital admission. Some states used inconsistent nomenclature for the resource being allocated by their triage procedure; these states were categorized based on the resource referenced in the section header accompanying the triage procedure.

### Prognostication Beyond Hospital Discharge

**Figure 3.**
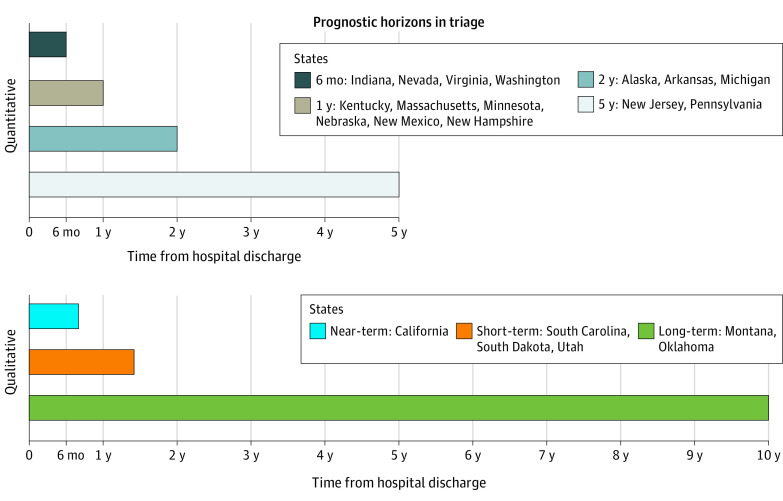
Prognostic Horizons in Triage Of the 32 state policies analyzed, most (21) called on triage officers to prognosticate survival beyond hospital discharge. Of these, 15 states called for prognostication to certain end points (6-month, 1-year, 2-year, 5-year). Six incorporated a prognostic assessment using only qualitative terms (near-term, short-term, long-term). Oklahoma and Montana instructed triage officers to consider long-term survival, specifying 10-year survival for the subset of patients with malignant neoplasm.

Most states analyzed (21 of 32 [66%]) incorporated estimates of survival beyond the acute hospitalization into triage procedures (Figure 2 and Figure 3). Most of these states (15 of 21 [71%]) stipulated discrete time horizons (eg, 6-month survival, 5-year survival). Predictions of survival 1 year beyond discharge were most common (6 of 15 [40%]), followed by 6 months (4 of 15 [27%]) and 2 years (3 of 15 [20%]). Two states (New Jersey and Pennsylvania) included prognostication to 5 years. The remaining 6 states only included descriptive terms of duration of survival beyond hospital discharge (eg, near-term; short-term). Two states (Montana and Oklahoma) incorporated “long-term” survival into their triage policies, instructing triage officers to consider 10-year survival for the subset of patients with malignant neoplasms.

## Discussion

### Heterogeneity in Resource Allocation

There was striking heterogeneity in the 32 state-promulgated written triage procedures that were publicly available for analysis in June 2023, particularly related to the role of chronic illness and postdischarge prognosis in triage. Even among the commonly listed conditions in triage procedures, there was little agreement on the degree of disease severity required before a patient’s access would be restricted or what parameters should be considered in assessing the disease’s severity. During triage, patients with moderate dementia would have reduced access to critical care in Nebraska and Montana; however, in Nevada, this would only apply to patients with dementia who are eligible for hospice. Within some states’ lists, conditions with significantly different prognoses factored equally into triage. In South Carolina, cardiac arrest unresponsive to conventional therapies and immunosuppression with high short-term mortality both categorically excluded a patient from intensive care unit admission. There was little clarity on the criteria states used to develop lists of conditions and proposed disease severity assessments for triage procedures. For instance, inclusion of cystic fibrosis with post-bronchodilator forced expiratory volume in the first second (FEV_1_) of less than 30% appears to have been derived from survival data from the 1970s and 1980s.^17^

Given states’ authority to regulate health care, policy heterogeneity is expected. Indeed, the National Academy of Medicine literature recommends extensive community engagement in the development of triage plans.^6,20^ However, the speed with which policies were developed or adapted for the COVID-19 pandemic suggests that debate and community consultation were unlikely.^21,22^ Instead, the varied approaches may reflect scientific uncertainty about predictors of survival and ethical disagreement about triage criteria, processes, and goals, such as whether the object of triage is to maximize life-years saved or save the greatest number of lives.^10,11,23^ This analysis demonstrates that this variation extends to how comorbidities should impact triage, whether triage officers should prognosticate beyond hospital discharge, and whether they can prognosticate potentially far into the future. While lists of comorbidities and a requirement for postdischarge prognostication could be viewed as tools to distinguish between severely ill patients and harmonize triage across health systems within a state, their use presents serious clinical challenges, raises ethical concerns, and may reflect varying levels of compliance with civil rights legal obligations.

### Clinical Implications

Assessing disease severity and prognosticating beyond discharge are challenging tasks when hospital staff are stretched by a public health crisis, so that time and resources are extremely limited. For instance, Kansas’s triage procedure, promulgated during this study period, now incorporates a 9-point mobility assessment, raising concerns about both feasibility and disability discrimination. Factoring disease severity assessments into triage presupposes that supporting data, such as pulmonary function tests or oncology records, or even accurate information about functional status, are readily accessible in a crisis. It also requires that triage officers have sufficient training to accurately synthesize these data in time-sensitive, high-acuity settings.

When triage officers are required to exercise prognostic judgement, particularly for diseases outside their specialty and for periods beyond the acute hospitalization, they are likely to make mistakes. Even in low-acuity settings, prognostic models are inaccurate and perform differently across diverse patient populations.^24,25,26,27^ Likewise, studies have consistently demonstrated prognostic imprecision from clinicians, even when working within their specialty or with patients nearing the end of life.^28,29^ This uncertainty grows the longer the time horizon, raising concerns over the accuracy of postdischarge prognostication in triage.^30,31^ Instruction from Montana and Oklahoma to prognosticate likelihood of cancer-related survival 10 years after hospitalization poses a difficult, if not clinically impossible, task. For written triage protocols to maximize lives saved during a crisis, they must be clinically operationalizable and based on current empirical evidence. A requirement to base triage on long-term prognosis fails this test.

### Ethical and Legal Implications

Our analysis revealed grounds for ethical and legal concern. First, the use of subjective criteria to deprioritize, or in some cases, exclude patients from accessing a scarce resource, critical care, or hospital admission could invite inconsistency and bias. Some states use subjective language for disease severity assessment (eg, dementia with “loss of ability to smile” or “requiring assistance with activities of daily living” as exclusion criteria) or for prognostication beyond hospital discharge (eg, short-term, long-term). Three states (Alaska, Arkansas, and Michigan) deprioritize patients with “severe underlying disease with poor long-term prognosis and/or ongoing resource demand (e.g., home oxygen dependent, dialysis dependent),” language modeled after Minnesota’s triage procedure, which has since eliminated this phrase. Chronic disease can attenuate a patient’s chance of surviving acute illness or injury, but triage procedures containing vague instructions invite medical professionals’ well-documented prejudices against persons with physical and intellectual disabilities and older adults.^32,33,34^

Moreover, language referencing resource intensity and long-term prognostic assessments (eg, 5 years, long-term) raises concerns of ageism and ableism. Resource utilization based on underlying disability and subjective severity assessments invite quality-of-life judgments in violation of civil rights protections, including the Americans with Disabilities Act, Section 504 of the Rehabilitation Act, and Section 1557 of the Patient Protection and Affordable Care Act.^13,14,15,35^ Disability and aging advocates assert that allocation strategies that maximize life-years saved through long-term prognostication devalue the lives of disabled and older persons.^17,36,37,38^ OCR’s February 2022 guidance advised that triage procedures not consider “likelihood of survival long after hospital discharge,” as it “may depend upon many factors and may be difficult to predict” and was “unlikely to be related to the effectiveness of the medical interventions being allocated.”^13^ OCR further cautioned that including long-term prognosis may “screen out individuals with disabilities from access to care.”^13^ State policies constraining prognostication to the near-term or short-term may be more in line with OCR recommendations, although the ambiguity of these terms could still allow inconsistent application based on patient age or disability. The use of categorical exclusion criteria and instructions for triage officers to prognosticate years into the future indicate that many states’ written triage procedures still do not comply with antidiscrimination guidance issued by the OCR in early 2022.^13^

Patients with chronic conditions that confer less-than-average life expectancy would be disproportionately affected by the use of lists of conditions and prognostic assessments beyond hospital discharge during triage. Such factors threaten to magnify preexisting inequities for populations with a higher chronic disease burden. Black, Hispanic, and American Indian or Alaskan Native populations have a significantly higher chronic disease burden than their non-Hispanic White counterparts, including conditions featuring prominently in states’ lists.^39,40,41,42^ Moreover, 2 of 3 persons aged 65 years and older have 2 or more chronic conditions.^41^ Persons living with physical and neurocognitive disabilities likewise experience a high prevalence of chronic conditions.^43,44,45^ This is particularly concerning for those whose comorbidities might be predictive of poor 1-year, 2-year, 5-year, or long-term survival, who would be deprioritized or excluded from lifesaving care in 13 states. Triage procedures using postdischarge prognosis and/or factoring in chronic conditions could systematically restrict access to care for millions of individuals that fall into one or more of these vulnerable patient groups.

### Limitations

This study has limitations. Twenty states did not have written triage procedures available for analysis. States may have issued or revised their plans after June 16, 2023. Tribal Nations were not included for analysis as preliminary searches did not identify policies promulgated by the 2 largest nations, Cherokee and Navajo. Federal health systems (eg, Indian Health Service, Veterans Health Administration) were not included in the analysis because their policies must be operational across multiple states.

## Conclusion

Written triage procedures influence access to and denial of lifesaving care during crises and therefore are fundamental to the public’s trust in the health care system. This study found that states’ triage procedures included varying lists of comorbidities and requirements for estimating postdischarge life expectancy. Triage based on heterogenous lists of comorbidities and prognostic assessments beyond hospital discharge risks violating this public trust; threatens to restrict access to critical care for vulnerable populations; and raises clinical, ethical, and civil rights concerns. States and policy makers should make their PPPs accessible to the public, revisit their COVID-19 triage procedures, and remove expansive lists of comorbidities and consideration of postdischarge survival. A national effort to develop model triage tools and OCR reexamination of published policies could help ensure alignment with civil rights protections.

## Notes

Supplement 1. **eTable 1. **Summary of Pandemic Preparedness Plans and Categorization **eTable 2. **Complete Tabulation of Chronic Conditions **eFigure.** Continuum of Resources Allocated in Triage

Supplement 2.
Data Sharing Statement
