# Supplementary material for: Triage Procedures for Critical Care Resource Allocation During Scarcity
Source: JAMA Netw Open. 2023 Aug 29;6(8):e2329688. doi: 10.1001/jamanetworkopen.2023.29688 (PMC10466166; doi:10.1001/jamanetworkopen.2023.29688)
Supplement: Supplement 1. — eTable 1. Summary of Pandemic Preparedness Plans and Categorization eTable 2. Complete Tabulation of Chronic Conditions eFigure. Continuum of Resources Allocated in Triage [file jamanetwopen-e2329688-s001.pdf]

Supplemental Online Content

Ennis JS, Riggan KA, Nguyen NV, et al. Triage procedures for critical care resource allocation during scarcity. *JAMA Netw Open.* 2023;6(8):e2329688. doi:10.1001/jamanetworkopen.2023.29688

eTable 1. Summary of Pandemic Preparedness Plans and Categorization

eTable 2. Complete Tabulation of Chronic Conditions

eFigure. Continuum of Resources Allocated in Triage

This supplemental material has been provided by the authors to give readers additional information about their work.

© 2023 Ennis JS et al. *JAMA Network Open.*

eTable 1. Summary of Pandemic Preparedness Plans and Categorization

State	Met Inclusion Criteria?	List Type[a]	Prognostication beyond hospital discharge	Resource allocated in triage	Link to access
**Alabama**	Excluded -- no written triage guidelines	n/a	n/a	n/a	https://www.alabamapublichealth.gov/cep/assets/alabamacscguidelines2020.pdf
**Alaska**	Included	Deprioritization/Example	2 yr	Specific resource	https://dhss.alaska.gov/dph/Epi/id/SiteAssets/Pages/HumanCoV/SOA_DHSS_CrisisStandardsOfCare.pdf
**Arizona**	Included	No list	No	Critical Care	https://www.azdhs.gov/documents/preparedness/emergency-preparedness/response-plans/azcsc-plan.pdf
**Arkansas**	Included	Deprioritization/Example	2 yr	Specific resource	https://www.healthy.arkansas.gov/images/uploads/pdf/ARCOVID-19_Crisis_Standards_of_Care.pdf
**California**	Included	Tiebreaker/Example	near-term	Critical Care	https://www.cdph.ca.gov/Programs/CID/DCDC/CDPH%20Document%20Library/COVID-19/California%20SARS-CoV-2%20Crisis%20Care%20Guidelines%20-June%208%202020.pdf
**Colorado**	Included	No list	No	Critical Care	https://drive.google.com/file/d/1SKT49ps1dxpPsByPr3z0QiISwmqvTUGM/view
**Connecticut**	Excluded -- no written triage guidelines	n/a	n/a	n/a	https://portal.ct.gov/-/media/Departments-and-Agencies/DPH/dph/legal/StandardsofCarefinalpdf.pdf
**Delaware**	Included	No list	No	Critical care	https://www.centerforpublicrep.org/wp-content/uploads/2020/05/DE-CSC-ConOps-FInal-4-29-20.pdf
**Florida**	Excluded -- no endorsement	n/a	n/a	n/a	https://fbn.miami.edu/_assets/pdf/resources/covid-19-resources/csc-fbn-6.pdf
**Georgia**	Excluded -- not found	n/a	n/a	n/a	n/a
**Hawaii**	Excluded -- no longer publicly available	n/a	n/a	n/a	n/a
**Idaho**	Included	Exclusion/Example	No	Critical care	https://coronavirus.idaho.gov/wp-content/uploads/2022/02/2022_ID_CSC_Standards_20220210.pdf

© 2023 Ennis JS et al. *JAMA Network Open.*

State	Met Inclusion Criteria?	List Type[a]	Prognostication beyond hospital discharge	Resource allocated in triage	Link to access
**Illinois**	Excluded -- no written triage guidelines	n/a	n/a	n/a	https://dph.illinois.gov/covid19/community-guidance/emergency-preparedness-hospitals.html
**Indiana**	Included	Exclusion/Example	6 mo	Specific resource	https://drive.google.com/file/d/12cdA1VRK27HhDLHsVJvTKKpE9-47txKV/view
**Iowa**	Excluded -- no written triage guidelines	n/a	n/a	n/a	http://publications.iowa.gov/17889/1/panflu_ehtical_guidelines_manual.pdf
**Kansas**	Included	Exclusion/Prescriptive<br><br>Kansas also listed deprioritizing conditions in their Chronic Advanced Organ Dysfunction Score	No	Critical Care	https://www.kdhe.ks.gov/DocumentCenter/View/29192/Kansas-Crisis-Standards-of-Care-Guidance-KCSCG-for-Hospitals-PDF
**Kentucky**	Included	Exclusion/Example	1 yr	Hospital Admission	https://www.kyha.com/assets/docs/COVID19/Update/CrisisStandardsofCareFinal.pdf
**Louisiana**	Included	Exclusion/Prescriptive	No	Hospital Admission	https://lsu.edu/ethics/files/crisis-standards-of-care-2019-protected.pdf
**Maine**	Excluded -- no written triage guidelines	n/a	n/a	n/a	http://themha.org/policy-advocacy/Issues/Novel-Coronavirus-(2019-nCoV)/Maine-Crisis-Standards-of-Care-Plan-0050317-v27-19.aspx
**Maryland**	Excluded -- not found	n/a	n/a	n/a	n/a
**Massachusetts**	Included	No list	1 yr	Critical care	https://www.centerforpublicrep.org/wp-content/uploads/crisis-standards-of-care-guidance-10-20-2020.pdf
**Michigan**	Included	Deprioritization/Example	2 yr	Specific resource	https://www.michigan.gov/-/media/Project/Websites/coronavirus/Folder2/MDHHS_Ethical_Guidlines_-_November_30_2021_FINAL_12-6-21.pdf?rev=1166ed3704944b6994bef3e188225dcf
**Minnesota**	Included	Deprioritization/Example	1 yr	Specific resource	https://www.health.state.mn.us/communities/ep/surge/crisis/standards.pdf
**Mississippi**	Excluded -- no written triage guidelines	n/a	n/a	n/a	https://msdh.ms.gov/msdhsite/index.cfm/44,8575,397,pdf/COVID-19_SOC_Plan_FY2022.pdf

© 2023 Ennis JS et al. *JAMA Network Open.*

State	Met Inclusion Criteria?	List Type[a]	Prognostication beyond hospital discharge	Resource allocated in triage	Link to access
**Missouri**	Excluded -- no endorsement	n/a	n/a	n/a	n/a
**Montana**	Included	Deprioritization/Example	long-term	Critical care	https://www.mthcc.org/assets/montana-crisis-care-guidance-final.pdf
**Nebraska**	Included	No list	1 yr	Critical care	https://dhhs.ne.gov/Documents/NE-Crisis-Protocol.pdf
**Nevada**	Included	Exclusion/Example	6 mo	Critical care	https://nvhealthresponse.nv.gov/wp-content/uploads/2020/07/NV_DHHS_DPBH_CSCRecommendations_COVID-19_071520_ADA.pdf
**New Hampshire**	Included	No list	1 yr	Specific Resource	https://www.dhhs.nh.gov/sites/g/files/ehbemt476/files/documents2/nh-crisis-standards-of-care-guidance.pdf
**New Jersey**	Included	No list	5 yr	Critical care	https://nj.gov/health/legal/covid19/AllocationMemoRevised.pdf
**New Mexico**	Included	Deprioritization/Prescriptive	1 yr	Specific resource	https://cv.nmhealth.org/wp-content/uploads/2021/11/Appendix-E.-Update-Triage-Protocol.pdf
**New York**	Included	Exclusion/Example	No	Specific resource	https://www.health.ny.gov/regulations/task_force/reports_publications/docs/ventilator_guidelines.pdf
**North Carolina**	Excluded – no longer publically available	n/a	n/a	n/a	n/a
**North Dakota**	Excluded -- no written triage guidelines	n/a	n/a	n/a	https://www.hhs.nd.gov/sites/www/files/documents/DOH%20Legacy/_nd-pandemic-influenza-plan-annex-to-the-state-public-health-plan.pdf
**Ohio**	Excluded -- no endorsement	n/a	n/a	n/a	https://ohiohospitals.org/OHA/media/OHA-Media/Documents/Patient%20Safety%20and%20Quality/COVID19/Ohio-Guidelines-for-Allocation-of-Scarce-Medical-Resources-CLEAN-FINAL.pdf
**Oklahoma**	Included	Deprioritization/Example	long-term	Specific resource	https://www.ok.gov/health2/documents/Hospital%20Crisis%20Standards%20of%20Care.pdf
**Oregon**	Included	No list	No	Critical care	https://sharedsystems.dhsoha.state.or.us/DHSForms/Served/le4019c.pdf

© 2023 Ennis JS et al. *JAMA Network Open.*

State	Met Inclusion Criteria?	List Type[a]	Prognostication beyond hospital discharge	Resource allocated in triage	Link to access
**Pennsylvania**	Included	No list	5 yr	Critical care	https://www.health.pa.gov/topics/Documents/Diseases%20and%20Conditions/COVID-19%20Interim%20Crisis%20Standards%20of%20Care.pdf
**Puerto Rico**	Excluded -- not found	n/a	n/a	n/a	n/a
**Rhode Island**	Included	Exclusion/Example	No	Critical care	https://health.ri.gov/publications/guidelines/crisis-standards-of-care.pdf
**South Carolina**	Included	Exclusion/Example	short-term	Critical care	https://scdhec.gov/sites/default/files/Library/CR-009538.pdf
**South Dakota**	Included	No list	short-term	Hospital Admission	https://sdaho.org/wp-content/uploads/2021/12/SD-Crisis-Standards-of-Care-FINAL_120921.pdf
**Tennessee**	Included	No list	No	Hospital Admission	https://www.tn.gov/content/dam/tn/health/documents/cedep/ep/Guidance_for_the_Ethical_Allocation_of_Scarce_Resources.pdf
**Texas**	Excluded -- not found	n/a	n/a	n/a	n/a
**Utah**	Included	Exclusion/Prescriptive	short-term	Critical care	https://www.utahhospitals.org/images/pdfs-doc/Utah_Crisis_Standards_of_Care_Guidelines_v9_12142020.pdf
**Vermont**	Included	Exclusion/Example	No	Specific resource	https://www.healthvermont.gov/sites/default/files/documents/pdf/Vermont-Crisis-Standards-of-Care-Plan-2021.pdf
**Virginia**	Included	No list	6 mo	Critical care	https://www.vdh.virginia.gov/content/uploads/sites/182/2021/01/Resource-Allocation-under-Crisis-Standards-of-Care-for-COVID.-FINAL.pdf
**Washington**	Included	Deprioritization/Example	6 mo	Specific resource	https://doh.wa.gov/sites/default/files/2022-02/821-151-CSC-TT-guidebook.PDF
**Washington, D.C.**	Excluded -- not found	n/a	n/a	n/a	n/a
**West Virginia**	Excluded -- not found	n/a	n/a	n/a	n/a
**Wisconsin**	Excluded -- not found	n/a	n/a	n/a	n/a
**Wyoming**	Excluded -- not found	n/a	n/a	n/a	n/a

© 2023 Ennis JS et al. *JAMA Network Open.*

[a] Example refers to lists of conditions that explicitly grant triage officers permission to exercise clinical judgement and consider additional conditions of similar severity. Prescriptive refers to lists of conditions that do not explicitly permit the consideration of additional conditions. Kansas listed exclusion and deprioritizing conditions; no other states had multiple lists serving different functions within triage.

© 2023 Ennis JS et al. *JAMA Network Open.*

eTable 2. Complete Tabulation of Chronic Conditions Used for Triage in State Pandemic Preparedness Plans

Organ System	Condition	Number of states	State (function within triage[a])	Proposed Severity Assessment
**Neurologic**	Chronic disorder of consciousness	4	CA (T), KS (D), NV (E), WA (D)	Persistent vegetative state; Modified Rankin Score >=5,
	Neurologic injury or event	9	IN (E), KS (E), LA (E), NV (E), SC (E), WA (D), ID (E), NY (E), RI (E)	No motor response to painful stimulus; high expected mortality; persistent coma (>72 hrs); GCS < 6; neurospecialist assessment of minimal chance of recovery; irreversible; low chance of survival; CT evidence of herniation; massive MCA or brainstem stroke
	Alzheimer's disease and dementia	7	LA (E), KS (D), MT (D), NV (E), NM (D), OK (D), VT (E)	limited speech ability; no independent ambulatory ability; cannot sit up without assistance; loss of ability to smile; loss of ability to hold up head independently; hospice eligible; FAST >=7; Moderate; with poor prior prognosis
	Neurodegenerative disease (e.g. ALS, MS, SMA, Parkinson's)	4	KS (D), KY (E), LA (E), NM (D)	Advanced, progressive; requiring assistance with ADLs or requiring chronic ventilator support
**Cardiovascular**	Heart failure	13	AR (D), CA (T), IN (E), KS (D) LA (E), MI (D), MN (D), MT(D), NM (D), OK (D), SC (E), VT (E), WA (D)	EF <30%; persistent ischemia unresponsive to therapy; non-reversible ischemia with pulmonary therapy; ACC/AHA Stage D; NYHA Class III, NYHA IV, NYHA II; evidence of frailty; with poor prior prognosis; EF 21-40%; LVAD; chronic inotrope utilization
	Pulmonary hypertension	4	CA (T), LA (E), KS (D), NM (D)	WHO Class IV; WHO Class III; moderate RV dysfunction
	Cardiac arrest	9	IN (E), KS (E), LA (E), NV (E), SC (E), VT (E), ID (E), NY (E), RI (E)	Unwitnessed arrest; recurrent arrest; arrest unresponsive to standard measures; trauma related arrest
	Coronary artery disease	5	MT (D), NM (D), OK (D), VT (E), WA (D)	Severe multivessel; symptomatic; not amenable to treatment; multiple stents placed and/or CABG
	Hypotension	2	NV (E), NY (E)	Unresponsive to fluid resuscitation and vasopressors
**Respiratory**	Chronic Lung Disease	12	AK (D), AR (D), CA (T), IN (E), KS (D), MI (D), MN (D), MT (D), OK (D), SC (E), VT (E), WA (D)	Home oxygen dependent; FEV1<20%; moderately severe; FEV1<25%, with poor prior prognosis; on NIPPV; frailty
	Cystic Fibrosis	4	AR (D), LA (E), MI (D), MN (D)	Home oxygen dependent
	Restrictive lung disease / pulmonary fibrosis	7	AR (D), LA (E), MI (D), MN (D), MT (D), NM (E), OK (D)	Home oxygen dependent; FVC <35%, moderately severe; total lung capacity < 60% predicted; PaO2 <55 mm Hg
	Obstructive lung disease/ COPD	7	AR (D), LA (E), MI (D), MN (D), MT (D), NM (E), OK (D)	Home oxygen dependent; moderately severe; FEV1 <25%; severe secondary pulmonary hypertension; FEV1 <20%

© 2023 Ennis JS et al. *JAMA Network Open.*

Organ System	Condition	Number of states	State (function within triage[a])	Proposed Severity Assessment
**Gastrointestinal/Hepatic**	Cirrhosis	13	AR (D), CA (T), IN (E), KS (D), LA (E), MI (D), MN (D), MT (D), NM (D), OK (D), SC (E), VT (E), WA (D)	Ascites; history of variceal bleeding; fixed coagulopathy; encephalopathy; MELD >=20; history of decompensation; Pugh > 9, Child Pugh's class C; Ineligible for treatment' with poor prior prognosis; MELD > 15
	Acute hepatic failure	2	AR (D), MN (D)	Hyperammonemia
**Renal**	End stage renal disease	10	AK (D), AR (D), IN (E), KS (D), LA (E), MI (D), MT (D), OK (D), SC (E), WA (D)	Dialysis dependent; patients < 75; eGFR<15
**Endocrine**	Diabetes	1	KS (D)	Type I or II with serious complications, e.g., amputation, myocardial infarction, or stroke.
**Neoplastic**	Solid Organ malignancy	3	AR (D), MI (D), MN (D)	Poor prognosis for recovery
	Hematologic malignancy	4	AR (D), CA (T), MI (D), MN (D)	Poor prognosis for recovery; resistant or progressive despite conventional initial therapy
	Metastatic malignancy	7	CA (T), KS (D), LA (E), NV (E), NM (D), VT (E), WA (D)	Expected survival <= 6 months; incurable; poor expected response to therapy
	Malignancy	5	MT (D), NM (D), OK (D), SC (E), WA (D)	Expected survival <10 years; ECOG >=3; palliative treatment only; advanced cancer; unresponsive to interventions
	Sum of the above, all states including malignancy	13	AR (D), CA (T), MI (D), MN (D), LA (E), NV (E), NM (D), VT (E), WA (D), OK (D), SC (E)	
**Injury**	Severe burns	8	IN (E), LA (E), NV (E), SC (E), VT (E), WA (D), ID (E), NY (E)	Predicted survival <=10%; BSA > 40%; inhalational injury; age >60*; <50% survival on triage for burn victims assessment; burn center assessment of minimal chance of survival; low chance of survival based on American Burn Association guide
	Trauma	5	LA (E), NV (E), VT (E), ID (E), UT (E)	RTS <2; with poor expected outcome
**General**	End stage organ failure	2	KY (E), VT (E)	poor prior prognosis
	Hospice eligible	4	AR (D), IN (E), LA (E), NV (E)	
	Immunosuppression	1	SC (E)	High short-term mortality
	Neonatal	1	MT (E)	Newborn with gestational age < 24 weeks; bilateral grade 4 intraventricular hemorrhage; total bowel loss due to necrotizing enterocolitis; birthweight < 500 grams

© 2023 Ennis JS et al. *JAMA Network Open.*

[a] Function within triage refers to role of the state's list within triage, either Exclusion (E), Deprioritization (D), or Tiebreaker (T). Kansas listed exclusion and deprioritizing conditions; no other states had multiple lists serving different functions within triage.

Abbreviations:

GCS=Glasgow Coma Scale; CT=computed tomography; MCA=middle cerebral artery; ALS-= amyotrophic lateral sclerosis; MS=multiple sclerosis; SMA=spinal muscular atrophy; FAST=Functional Assessment Scale Tool; ADLs=activities of daily living; EF=ejection fraction; ACC/AHA=American College of Cardiology/American Heart Association; NYHA=New York Heart Association; LVAD=left ventricular assist device; WHO=World Health Organization; RV=right ventricle; CABG=coronary artery bypass graft; FEV1= forced expiratory volume; NIPPV=non-invasive positive pressure ventilation; FVC=forced vital capacity; TLC=total lung capacity; COPD=chronic obstructive pulmonary disease; MELD=Model for End Stage Liver Disease; eGFR=estimated glomerular filtration rate; ECOG=Eastern Cooperative Oncology Group; BSA=body surface area; RTS=Revised Trauma Score

© 2023 Ennis JS et al. *JAMA Network Open.*

eFigure. Continuum of Resources Allocated in Triage

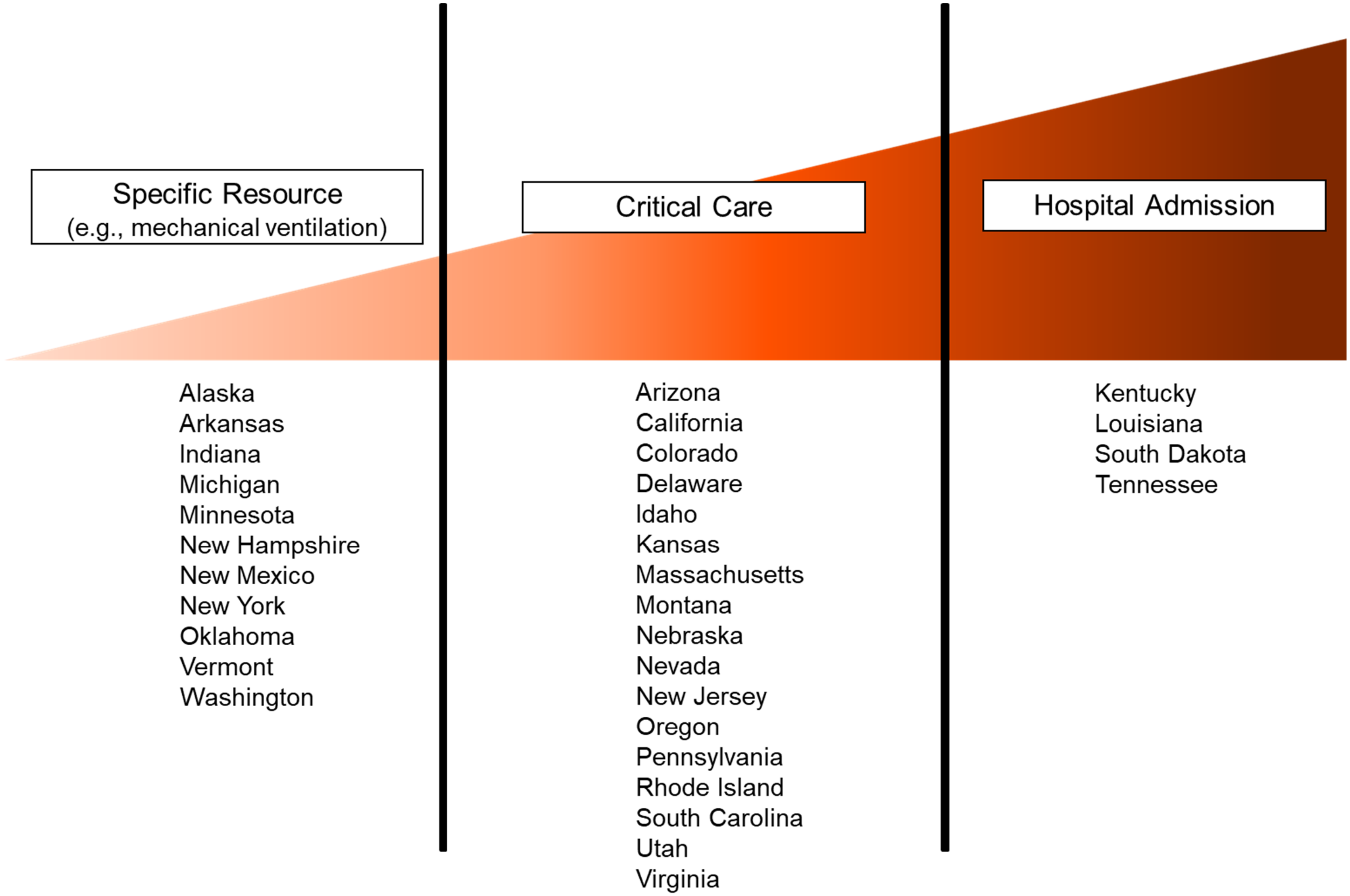

© 2023 Ennis JS et al. *JAMA Network Open.*
