# Supplementary material for: Triage Procedures for Critical Care Resource Allocation During Scarcity
Source: JAMA Netw Open. 2023 Aug 29;6(8):e2329688. doi: 10.1001/jamanetworkopen.2023.29688 (PMC10466166; doi:10.1001/jamanetworkopen.2023.29688)
Supplement: Supplement 2. — Data Sharing Statement [file jamanetwopen-e2329688-s002.pdf]

Data Sharing Statement

Ennis. Triage Procedures for Critical Care Resource Allocation During Scarcity. *JAMA Netw Open*. Published August 24, 2023. doi:10.1001/jamanetworkopen.2023.29688

Data
Data available: Yes
Data types: Other (please specify)
Additional Information: Data is available publicly via the links to the policies analyzed in eTable 1.
How to access data: Data is available publicly via the links to the policies analyzed in eTable 1. For further information or access, contact demartino.erin@mayo.edu.
When available: With publication

Supporting Documents
Document types: None

Additional Information
Who can access the data: Data will be made available to individuals with a signed data use agreement, per Mayo Clinic policy.
Types of analyses: Any purpose accompanied by a signed data use agreement.
Mechanisms of data availability: With a signed data use agreement, per Mayo Clinic policy.
